# Semi-Quantitative Assay to Measure Urease Activity by Urinary Catheter-Associated Uropathogens

**DOI:** 10.3389/fcimb.2022.859093

**Published:** 2022-03-22

**Authors:** Jesus M. Duran Ramirez, Jana Gomez, Chloe L. P. Obernuefemann, Nathaniel C. Gualberto, Jennifer N. Walker

**Affiliations:** ^1^ Department of Microbiology and Molecular Genetics, McGovern Medical School, The University of Texas Health Science Center, Houston, TX, United States; ^2^ Department of Epidemiology, Human Genetics, and Environmental Sciences, Center for Infectious Diseases, School of Public Health, The University of Texas Health Science Center, Houston, TX, United States; ^3^ The Center for Women’s Infectious Disease Research, Department of Molecular Microbiology, Washington University School of Medicine, St. Louis, MO, United States

**Keywords:** urease, urease detection assay, urease-producing uropathogens, CAUTI, *Staphylococcus aureus*, *Proteus* spp., *Klebsiella* spp., *Morganella* spp.

## Abstract

Catheter-associated urinary tract infections (CAUTIs) are one of the most common healthcare-associated infections in the US, accounting for over 1 million cases annually and totaling 450 million USD. CAUTIs have high morbidity and mortality rates and can be caused by a wide range of pathogens, making empiric treatment difficult. Furthermore, when urease-producing uropathogens cause symptomatic CAUTI or asymptomatic catheter colonization, the risk of catheter failure due to blockage increases. The enzyme urease promotes catheter blockage by hydrolyzing urea in urine into ammonia and carbon dioxide, which results in the formation of crystals that coat the catheter surface. If CAUTI is left untreated, the crystals can grow until they block the urinary catheter. Catheter blockage and subsequent failure reduces the quality of life for the chronically catheterized, as it requires frequent catheter exchanges and can promote more severe disease, including dissemination of the infection to the kidneys or bloodstream. Thus, understanding how urease contributes to catheter blockages and/or more severe disease among the broad range of urease-producing microbes may provide insights into better prevention or treatment strategies. However, clinical assays that detect urease production among clinical isolates are qualitative and prioritize the detection of urease from *Proteus mirabilis*, the most well-studied uropathogenic urease producer. While urease from other known urease producers, such as *Morganella morganii*, can also be detected with these methods, other uropathogens, including *Staphylococcus aureus* and *Klebsiella pneumonia*, are harder to detect. In this study, we developed a high throughput, semiquantitative assay capable of testing multiple uropathogens in a rapid and efficient way. We validated the assay using Jack Bean urease, the urease producing species*: Proteus* spp.*, M. morganii, K. pneumonia*, and *S. aureus* strains, and the non-urease producer: *Escherichia coli*. This modified assay more rapidly detected urease-producing strains compared to the current clinical test, Christensen Urea Agar, and provided semiquantitative values that may be used to further investigate different aspects of urease regulation, production, or activity in these diverse species. Furthermore, this assay can be easily adapted to account for different environmental stimuli affecting urease production, including bacterial concentration, aeration, or addition of anti-urease compounds.

## Introduction

More than 30 million urinary catheters are placed annually in the US, and of these, 10-30% become infected (Darouiche, 2001; Trautner and Darouiche, 2004; Nicolle, 2012; Flores-Mireles et al., 2015; Klein and Hultgren, 2020). These high infection rates result in more than 1 million cases annually, making catheter-associated urinary tract infection (CAUTI) the most common hospital-associated infection in the US (Ronald, 2003; Trautner and Darouiche, 2004; Cope et al., 2009; Trautner, 2010; Nicolle, 2012; Flores-Mireles et al., 2015; Chenoweth and Saint, 2016). With an annual cost of 450 million USD, which as of 2008 is no longer reimbursable under the Center for Medicare and Medicaid Services, CAUTI prevention has become a priority (Peasah et al., 2013; Meddings et al., 2014). However, prevention and treatment of CAUTI remains challenging for several reasons. First, urinary catheters increase the range of pathogens capable of causing urinary tract infections (UTIs), which makes empiric treatment difficult (Ronald, 2003; Trautner and Darouiche, 2004; Nicolle, 2012; Flores-Mireles et al., 2015; Pelling et al., 2019). Specifically, while UTIs in normal healthy individuals are primarily caused by *Escherichia coli* (>80%), no single pathogen accounts for >25% of all CAUTIs (Wagenlehner et al., 2012; Weiner et al., 2016). Furthermore, while the majority of pathogens causing CAUTI only account for 2-10% of infections each, that translates to thousands of cases caused by each species annually. Second, CAUTI pathogens form recalcitrant biofilms on the catheter surface that impair antibiotic activity (Trautner and Darouiche, 2004; Nicolle, 2012; Flores-Mireles et al., 2015). Additionally, increasing antibiotic resistance among uropathogens further complicates treatment options. Recent work shows that empiric antibiotic treatment of CAUTI fails to improve patient outcomes and may result in additional complications, such as the development of *Clostridium difficile* diarrhea and/or antibiotic resistance (Babich et al., 2017). Lastly, urinary catheters increase the rate of symptomatic UTI and asymptomatic bacteriuria (ASB), with chronically catheterized individuals at highest risk (Flores-Mireles et al., 2015; Nicolle, 2019).

**Figure 1:**
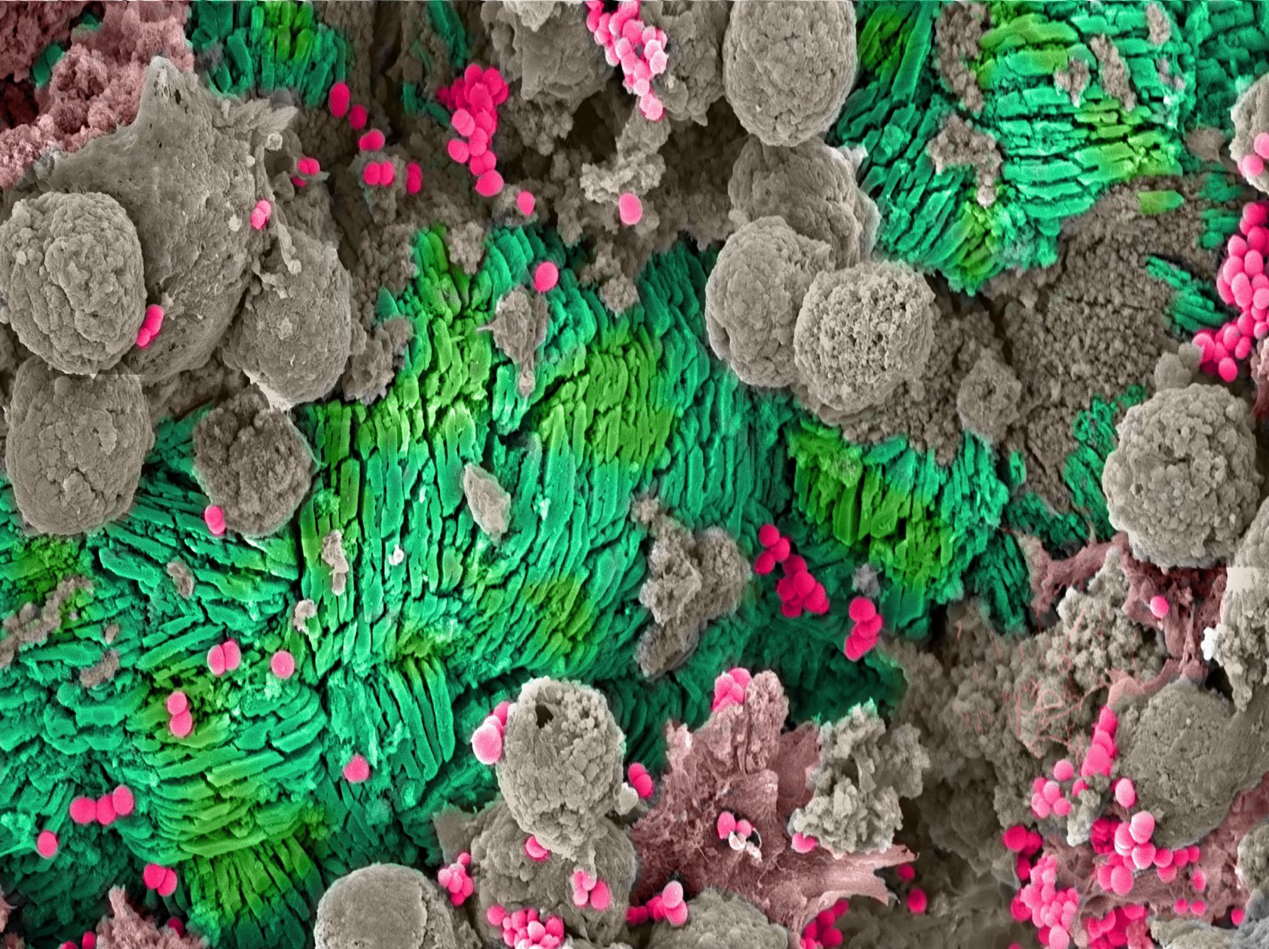
Catheter Encrustation. Scanning electron microscope (SEM) image of crystalline precipitates forming a catheter encrustation (green) resulting from asymptomatic catheter-associated bacteriuria caused by *Staphylococcus aureus* (pink). The encrustation and bacteria are surrounded by host immune cells (gray).

For individuals with chronic indwelling urinary catheters, ASB can also lead to catheter blockages, which decreases the quality of life for these individuals as it results in frequent device failure and increases the risk of additional complications, such as the development of pyelonephritis and bacteremia (Warren et al., 1994; Broomfield et al., 2009; Stickler and Feneley, 2010). Catheter blockages are typically caused by encrustations that form following infection with CAUTI pathogens that encode the enzyme urease, including *Proteus* spp.*, Providencia* spp.*, Klebsiella pneumoniae*, *Morganella morganii*, and *Staphylococcus aureus* (Hedelin, 2002; Broomfield et al., 2009). Specifically, urease hydrolyzes urea, present in urine, into ammonium and carbon dioxide, which in turn, drastically increases the pH of urine and results in crystalline precipitate formation. Accumulation of crystalline precipitates on the catheter surface leads to urinary catheter encrustations **(** **Figure 1** **)** and, if the infection is left untreated, can result in catheter blockages and failure of the device (Gatermann et al., 1989; Gatermann and Marre, 1989; Stickler and Feneley, 2010; Konieczna et al., 2012; Armbruster et al., 2014; Armbruster et al., 2017). With urinary catheter use expected to increase as the population ages, CAUTI, and therefore catheter blockages, are likely to become more common (Trautner, 2010). Thus, greater insights into the mechanisms that promote CAUTI with urease producing pathogens are urgently needed.

Urease is a highly conserved, nickel-containing enzyme that is composed of three subunits, subunit α, subunit β, and subunit γ, and multiple accessory proteins. The gene *ureC* encodes the active site (subunit α), which hydrolyzes urea. Together with the genes encoding *ureA* (subunit γ) and *ureB* (subunit β), the α, β and γ subunits form the apoenzyme, which is inactive. The additional accessory genes, *ureD*, *ureE*, *ureF*, *ureG*, and *ureH*, which encode additional metal coordination sites, are needed to form the fully-functioning holoenzyme and enable urease activity (Konieczna et al., 2012). Despite being highly conserved across many CAUTI pathogens, only the urease encoded by *Proteus mirabilis* is well characterized among urease-producing uropathogens (Broomfield et al., 2009; Kosikowska and Berlicki, 2011; Konieczna et al., 2012; Armbruster et al., 2014). Studies investigating the *P. mirabilis* enzyme have shed light on how urease not only contributes to catheter encrustations, but also irritates the mucosal epithelium, exacerbates inflammation, and provides additional surfaces for biofilm formation (Gatermann et al., 1989; Gatermann and Marre, 1989; Stickler and Feneley, 2010; Konieczna et al., 2012; Armbruster et al., 2014; Armbruster et al., 2017). Additional studies dissecting the regulation of *P. mirabilis* urease indicate that the transcriptional activator UreR, which is encoded directly upstream of the operon, is induced by the presence of urea (Nicholson et al., 1993; Dattelbaum et al., 2003; Konieczna et al., 2012). Thus, when *P. mirabilis* is grown in the presence of urea, urease is expressed. Notably, while probe-based DNA-hybridization assays and bioinformatics analyses indicate that other *Proteus spp*, including *P. vulgaris* and *P. rettgeri*, and *Providencia* spp., such as *P. stuartii*, also encode *ureR* homologues, other urease-producing CAUTI pathogens, like *M. morganii*, *S. aureus*, and *K. pneumoniae*, do not (Dattelbaum et al., 2003; Konieczna et al., 2012). Recent work suggests *M. morganii* urease expression is constitutive, while *K. pneumoniae* urease may be controlled through the nitrogen regulatory system (Hu et al., 1990; Collins et al., 1993; Nicholson et al., 1993; Dattelbaum et al., 2003). Additionally, previous studies suggest several regulators may control *S. aureus* urease expression, including the global regulators CodY and CcpA, as well as the Agr quorum sensing system (Seidl et al., 2009; Zhou et al., 2019). Thus, these studies not only indicate that urease is regulated differently within these uropathogens, but they also suggest that the current activity assays used to detect urease may fail to account for the different signals needed to activate the expression, production, or activity of urease within these diverse species.

Current clinical urease assays are qualitative and include the Christensen Urea Agar method, primarily used for detection among uropathogens, as well as the rapid urease test (RUT), which is typically used for *Helicobacter pylori*, a bacterium that infects the stomach (Christensen, 1946; Brink, 2013; Uotani and Graham, 2015). Briefly, the Christensen Urea Agar method is performed by streaking a single bacterial colony onto an agar slant or plate that contains urea, glucose, peptone, salts, and a pH indicator (phenol red) (Christensen, 1946). The slant is then incubated at 37°C and observed for up to 6 days. For urease producing bacteria, the hydrolysis of urea within the agar by the enzyme causes an increase in pH, which results in a color change due to the presence of the pH indicator in the media. Thus, this assay identifies inducible or constitutive urease producers, like *Proteus* spp. and *M. morganii*, within a few hours of incubation. However, false positives can result after long incubation periods due to the production of growth byproducts that also change the pH of the media, thus a non-inoculated or a known non-urease producer control slant is required to account for any non-urease specific color change of the media. Similarly, the RUT assay, which is primarily used to detect *H. pylori* urease, is performed by placing the bacteria in RUT media, which contains an antibacterial agent, urea, and a pH indicator. The sample is then incubated at 38°C for no more than 24 hours (Uotani and Graham, 2015). Here again, if a urease producer is present, urea hydrolysis results in a color change of the media due to the pH indicator. The RUT urease test is dependent on the initial concentration of the bacteria used, and similarly to the Christensen Urea Agar method, can also be affected by other bacterial byproducts that change the pH of the media. Specifically for the RUT media, any color change after the 24 hour time limit is considered a false positive (Uotani and Graham, 2015). Thus, these assays are adept at identifying high urease producers, generally with constitutive or urea inducible expression, like *Proteus* spp.*, M. morganii*, or *H. pylori*, and provide qualitative results. However, for other urease-producing uropathogens that do not encode the *ureR* gene, which upregulates urease production in the presence of urea, such as *S. aureus* and *K. pneumonia*, these assays are not as accurate at detecting urease. Specifically for *S. aureus*, the Christensen Urea Agar method only detects urease production in 25-50% of clinical isolates, yet bioinformatics analyses suggest the operon is present in >90% of all *S. aureus* strains sequenced and annotated in NCBI as of 2012 (Bichler et al., 2002; Gad et al., 2009; Konieczna et al., 2012; Flannigan et al., 2014). Furthermore, in addition to these clinical assays, phenol red-based broth assays have also recently been developed to provide semi-quantitative methods that allow researchers to further dissect urease regulation, expression, and activity (Richmond and Yep, 2019; Brauer et al., 2020; Sigurdarson et al., 2020). These broth-based assays rely on detecting the optical density (OD) at a single wavelength (between OD_550_ and OD_570_) to determine the color change of the media, which provides semi-quantitative values for urease activity. However, these methods were primarily developed to assess urease activity among individual pathogens that are generally considered high urease producers, like *P. mirabilis*. Notably, bacterial species without urea inducible or constitutively active urease enzymes, such as *S. aureus*, remain untested in these assays (Richmond and Yep, 2019; Brauer et al., 2020; Sigurdarson et al., 2020). It, therefore, remains unclear if these newly developed assays can be used to semi-quantitatively detect urease among all pathogens that produce the enzyme, especially considering these methods use a single OD wavelength, which may also be affected by bacterial growth or the production of growth byproducts that alters the color change of the media, similarly to the Christensen Urea Agar Assay.

Here, we set out to develop a high throughput, adaptable assay to more rapidly detect and quantify urease produced by diverse species, as well as to account for a wider range of culture conditions than the previously developed tests. Specifically, we adapted the Christensen Urea Agar assay to a 96 well plate broth assay, with the color change detected by a microtiter plate reader. The microtiter plate allows for the collection of semiquantitative values that are associated with urease specific color change caused by the increase in pH of the media. This adapted assay allowed for the easy manipulation of culture conditions, such as varied bacterial concentration and aeration, as well as the use of additional controls, including the use of media with and without urea, for more accurate detection of urease production in a broader range of uropathogens. Using this assay, we assessed a diverse set of CAUTI pathogens for urease production, including *Proteus* spp.*, M. morganii, S. aureus, K. pneumoniae*, and *E. coli.* We found that increasing concentrations of bacteria at the start of the assay correlated with quicker detection of urease activity among all strains tested, except for the non-urease producer, *E. coli*, which remained negative for urease. Furthermore, urease activity among all strains and species tested was detected more quickly in the adapted assay compared to the Christensen Urea Agar method. Notably, we found that urease could be detected among all of the *S. aureus* urinary catheter-associated isolates (7/7), regardless of the assay used. However, urease activity was again detected more quickly with our adapted assay (5-10 hours on average) compared to the Christensen Urea Agar assay (>24 hours). Together, this data suggests urease production and activity among *S. aureus* urinary catheter-associated isolates may differ from other non-UTI related *S. aureus* strains. Thus, this work demonstrates that the modified urease detection assay is an adaptable, high throughput method that can rapidly detect urease activity among a diverse array of urease-producing CAUTI pathogens, which can be used in future studies to investigate urease expression, production, and activity, and may even be adapted to explore therapeutics with inhibitory capabilities.

## Materials And Methods

### Bacterial Strains and Growth Conditions

All strains used in this study are listed in **Table 1**. All human urinary catheter (HUC) clinical isolates, including strains of *S. aureus* and *M. morganii*, were isolated from individuals with chronic indwelling urinary catheters as previously described (Walker et al., 2017; Walker et al., 2020). The *Escherichia coli* strain was isolated from a woman who experienced a UTI (Chen et al., 2009). The *K. pneumonia* strain was isolated from the respiratory tract of an asymptomatic individual and provided by Drs. Cesar Arias and Blake Hanson (Shropshire et al., 2021). The *Proteus* spp. strain was isolated from a mouse urinary catheter during experimental urinary catheterization. Brain heart infusion (BHI) broth (BD, cat # 237200) and BHI agar were used to maintain all cultures and prepare overnight cultures for experiments. All transposon mutants were generated using tryptic soy broth (Sigma; cat # C1016-500G) and maintained in BHI supplemented with 10 ug/mL erythromycin (Fisher; cat # AC227330050). The *ureC* clean deletion was generated in *Escherichia coli* under selection with 100 ug/ml ampicillin (Sigma, cat #BP1760-25G) in LB (Sigma; cat #BP1426-500) and then maintained in *S. aureus* under selection with 20 ug/ml chloramphenicol (Sigma; cat # CO37825G) in BHI. Once generated in the UTI MRSA background, the *ureC* clean deletion was maintained in BHI without antibiotic selection. Christensen Urea Agar (Sigma, cat # 27048-500G-F) plates and slants supplemented with filter sterilized 40% urea solution were used according to the manufactures protocol to determine urease activity. Briefly, slants and plates were inoculated with a single colony from a BHI agar plate and incubated at 37°C for 50 hours. For adapted urease activity broth assays, BHI broth was inoculated with a single colony from a BHI agar plate and grown, shaking at 175 rpm for at least 18 hours at 37°C. Bacterial strains were then either *i*) spun down at 10,000 g and resuspended or *ii*) diluted to various concentrations in urease broth. Urease broth consisted of 1 g peptone (BD; cat # 211921), 1 g D(+)-glucose (Sigma; cat #G8270-1KG), 2 g potassium phosphate monobasic (Sigma; cat # P9791-100G), 5 g sodium chloride (Fisher; cat # S671-500), 0.012 g Phenol red (Sigma; cat # 114529-5G), in 1 L of MilliQ water, pH 6.8. Following sterilization *via* autoclaving, urease broth was either supplemented with filter sterilized 40% urea solution (Fisher; cat # BP169-500) or with an equivalent amount of sterile water (the solution urea was dissolved in). Urease broth with and without urea allowed the determination of urease-specific activity across diverse bacterial species. Potassium phosphate monobasic was a key component in the broth. When lower grade or a different type of phosphate was used, the timing and/or the intensity of the color change of the media was altered. Since this assay relies on a pH change in the media following urea hydrolysis by urease, the buffering capacity of each phosphate may contribute to any difference observed.

**Table 1:** Strain list.

Strain Name	Isolation site	Genus	Species	Citations
**JE2**	skin infection	*Staphylococcus*	*aureus*	Bae et al., 2008
**JE2 *ureC::tn***	skin infection	*Staphylococcus *	*aureus*	This Study
**UTI MRSA**	Urine	*Staphylococcus*	*aureus*	Walker et al., 2017
**UTI MRSA *ureC::tn***	Urine	*Staphylococcus*	*aureus*	This Study
**UTI MRSA Δ*ureC***	Urine	*Staphylococcus*	*aureus*	This Study
**HUC 86**	Urinary Catheter	*Staphylococcus*	*aureus*	This Study
**HUC 86-02**	Urinary Catheter	*Staphylococcus*	*aureus*	This Study
**HUC 95**	Urinary Catheter	*Staphylococcus*	*aureus*	This Study
**HUC 97-02**	Urinary Catheter	*Staphylococcus*	*aureus*	This Study
**HUC 100**	Urinary Catheter	*Staphylococcus*	*aureus*	This Study
**HUC 100-01**	Urinary Catheter	*Staphylococcus*	*aureus*	This Study
**HUC 111-01C**	Urinary Catheter	*Staphylococcus*	*aureus*	This Study
**C699**	Respiratory tract	*Klebsiella*	*pneumonia*	Shropshire et al., 2021
**HUC 87**	Urinary Catheter	*Morganella*	*morganii*	This Study
**JNW153**	Urinary Catheter*	*Proteus spp*		This Study
**DH5α**	Cloning strain	*Escherichia*	*coli*	Invitrogen cat # LS18265017
**RN4420**	Cloning strain	*Staphylococcus*	*aureus*	Walker et al., 2013
**UTI89**	UTI	*Escherichia*	*coli*	Chen et al., 2009

A total of 15 strains representing four species were tested to validate the adapted urease activity assay.

*mouse catheter.

### Scanning Electron Microscopy Image of Catheter Encrustation

The human urinary catheter was previously collected and clinical characteristics were published (catheter #7) (Walker et al., 2020). Scanning electron microscopy (SEM) was performed as previously described (Walker et al., 2017; Walker et al., 2020). Briefly, a 3 cm long piece of the tip of the urinary catheter was fixed in 10% neutral buffered formalin, washed in 0.15 M sodium cacodylate buffer, and then stained with 1% osmium tetroxide. Next, a graded ethanol series (50%, 70%, 90%, 100%, and 100%) was used to dehydrate the sample. A critical point drier (Leica CPD 300, Vienna Austria) was used to dry the sample, which was then sputter-coated with 6 nm iridium (Leica ACE 600, Vienna, Austria). A Zeiss Merlin FE-SEM equipped with a Gemini II electron column (Oberkochen, Germany) was used to image the catheter with secondary electrons detected by the Everhart-Thornley secondary electron detector at an accelerating voltage of 3 keV and a beam current of 200nm.

### Urease Carriage Among *S. aureus* Strains

Bioinformatics analyses were used to determine the percentage of sequenced *S. aureus* isolates in NCBI that carry *ureC*, which was used as a marker for the carriage of the urease operon. A total of 10,290 *S. aureus* assemblies downloaded from NCBI were used. Briefly, open reading frames were identified for each assembly using Prokka v1.14.5 and all open reading frames were clustered using Roary v3.13.0 (Seemann, 2014; Page et al., 2015). The open reading frame encoding *ureC* was then identified by sequence match to the *ureC* gene in *S. aureus* strain JE2, and the presence-absence matrix from Roary was used to calculate prevalence.

### Urease Carriage Among HUC Isolates

To confirm that the *S. aureus* HUC strains encode urease, genomic DNA was extracted using the Promega Miniprep Kit (Promega; cat # A1330) according to the manufacturer’s guidelines, with the exception that 2 ug/ml of Lysostaphin (Sigma; cat#) was added to the cells resuspended in P1 buffer and incubated for 1 hr at 37°C to digest the staphylococcal cell wall. PCR was then used to confirm the presence of the *ureC* gene, which encodes the active site within the urease enzyme, in the genome. The ureC-Forward and ureC-Reverse primers shown in **Table 2** were created based on the JE2 genome downloaded from the NCBI database. The urease operon of *K. pneumonia* C699 was confirmed *via* genomic analysis, previously performed in (Shropshire et al., 2021).

**Table 2:** Primers used in this study.

Primer Name	Sequence (5’-3’)	Citations
ureC-Forward	ATGAGCTTTAAAATGAACG	This Study
ureC-Reverse	ATGTTCCTCCTAGAATAAG	This Study
ureC-Forward-Mutant check	GACGCAAAATCAATATACGAGCTT	This Study
ureC-Reverse-Mutant check	CTTCATATGTTTGTGGATCAACG	This Study
Buster primer	GCTTTTTCTAAATGTTTTTTAAGTAAATCAAGTAC	Bae et al., 2008
ureC KO upstream for	ccgaattcGATTACAGATATCGAAATCGAGGCTA	This Study
ureC KO upstream rev	TCATGATCTTTTTCCTCCTTTTTTATTCAC	This Study
ureC KO downstream linker	GAATAAAAAAGGAGGAAAAAGATCATGAGAGGAACATAGAATGATTGTTGAAG	This Study
ureC KO downstream rev	ccgtcgacCCTGTTGGAAACTGTGAATCACAG	This Study

Upper case sequences denote the genomic regions amplified based on the JE2 sequence obtained from NCBI and lower case sequences denote the restriction enzyme sites EcoRI and SalI.

### Generation of the Urease Transposon Mutant Strains

The transposon mutant in *ureC* (*ureC::tn*) was obtained from the Nebraska Transposon Mutant Library (BEI Resources). UTI MRSA *ureC::tn* and JE2 *ureC::tn* mutants were generated *via* transduction of the wildtype strains with lysates made from the Nebraska Transposon Mutant Library *ureC::tn* mutant strain using phage 11, as previously described (Walker et al., 2017). Briefly, a *ureC::tn* mutant lysate was generated by combining the Nebraska Transposon Mutant Library strain containing the *ureC* transposon mutation with phage 11 in melted top agar and transferring the solution to a TSA plate containing 5 mM calcium chloride. The top agar was allowed to cool and the plates were incubated at 37°C overnight. Plates containing 30-300 plaque forming units were selected, top agar was removed, and plaques were extracted *via* centrifugation followed by purification with a 0.22 um syringe filter (Sigma; cat # SLVGM33RS). The resulting phage lysate was then used to introduce the *ureC* transposon mutation into the UTI MRSA and JE2 background. For this, the UTI MRSA and JE2 strains were streaked on a TSA slant and incubated overnight to create a lawn. The bacteria were then resuspended in 1 mL TSA supplemented with 5 mM calcium chloride. The purified *ureC::tn* mutant phage lysate and the resuspended bacteria were then incubated, shaking for 20 minutes at 37°C. Cold, sterile 20 mM sodium citrate (Sigma; cat # W302600-1KG-K) was then added to these cultures, which were then spun down, resuspended in 20 mM sodium citrate (Sigma; cat # W302600-1KG-K), and 100 ul was spread on TSA plates containing 20 mM sodium citrate and erythromycin. Colonies that grew were restreaked on TSA with sodium citrate and erythromycin to select against any contaminating phage 11 and to select for the *ureC::tn* mutation, respectively. DNA was extracted as described above and PCR was performed using the ureC-Forward-Mutant check and Buster primers listed in **Table 2** to confirm the presence of the *ureC::*tn mutation (UTI MRSA *ureC::tn* and JE2 *ureC::tn*).

### Generation of the *ureC* Clean Deletion Strain

The clean *ureC* deletion in the UTI MRSA background (UTI MRSA Δ*ureC*) was generated *via* splicing overlap extension (SOEing) PCR and homologous recombination, as previously described (Walker et al., 2013). Briefly, ~ 500 base pairs upstream and downstream of the *ureC* gene were amplified from the chromosomal DNA of UTI MRSA using the ureC KO primers listed in **Table 2**. Care was taken to ensure the deletion was inframe and did not disrupt the downstream genes of the rest of the urease operon. The upstream and downstream amplicons were then PCR purified using the Qiagen PCR purification kit (Qiagen; cat # 28106) and the *ureC* KO upstream for and *ureC* KO downstream rev primers containing the EcoRI and SalI restriction sites, respectively, listed in **Table 2**, were used in a SOEing PCR to generate a single amplicon. The single amplicon was then PCR purified and cloned into the TOPO-PCR 2.1 (Thermo Fisher; cat # K45002) according to the manufacturer’s protocol. The TOPO-PCR 2.1 *ureC* KO construct was then transformed into DH5α chemically competent cells according to the manufacturer’s protocol. The *ureC* KO construct that was generated was then digested with EcoRI and SalI and ligated into the plasmid pJB38, which was digested with the same restriction enzymes, as previously described (Walker et al., 2013). The ligation (pJB38 Δ*ureC*) was transformed into chemically competent DH5α, selecting on LB ampicillin, and colonies were confirmed for the correct construct using PCR and sanger sequencing. Plasmid pJB38 Δ*ureC* was extracted and transformed into electrocompetent *S. aureus* cells (RN4220), selecting on BHI chloramphenicol at 30°C. Electrocompetent cells were created by subculturing RN4420 to an OD_600_ of 0.05 in B2 media, which consists of 1% casein hydrolysate (Oxoid; cat # LP0041), 2.5% yeast extract (Fisher; BP1422-500), 0.5% glucose (Sigma; cat #G8270-1KG), 2.5% NaCl (Fisher; cat# S5671-500), and 0.1% K2HPO4 (Sigma; cat # P9791-100G). The subculture was then incubated for 2.5 hours, shaking at 37°C to an OD_600_ of 0.3-0.4. The cells were then harvested *via* centrifugation (ThermoSientific SORVALL LEGEND X1R) at 5,000 rpm for 10 mins at 4°C and washed with decreasing amounts (1 volume, ½ volume, and ¼ volume) of ice cold, sterile water. The final wash was performed with 1/10 volume ice cold 10% glycerol and the cells were resuspended in 1/250 volume 10% glycerol. Electrocompetent cells were then stored at -80°C until ready for use. Once electrocompetent UTI MRSA cells were created, they were transformed with plasmid pJB38 Δ*ureC* extracted from RN4220. Transformants were selected at 42°C overnight on BHI chloramphenicol. Colonies were screened for double recombination and loss of the plasmid *via* PCR using the ureC KO upstream forward and ureC KO downstream reverse primers listed in **Table 2** and for sensitivity to chloramphenicol, respectively. Sanger sequencing was used to confirm the mutation.

### Adapted Urease Activity Assay

For the adapted urease broth activity assay, strains from overnight cultures were either *i)* subcultured (1:100 or 1:1000) into urease broth with and without urea or *ii)* harvested by centrifugation for 5 minutes at 10,000 g, washed with 1 volume of 1X Phosphate Buffered Saline (PBS), and resuspended in urease broth with or without urea. Each strain was transferred to a 96-well plate and tested in triplicate with at least two biological replicates. Purified Jack Bean urease (Sigma; cat # 94281-1G), strains grown in urease broth without urea, urease broth alone with and without urea, and strains with mutants in urease were included as controls. The OD was measured at 415 nm, 560 nm, and 600 nm every 20 mins for 24 hrs, shaking at 37°C using a BioTek Synergy|H1 microtiter plate reader (BioTek, Vermont). The ratio of the OD detected at 560 nm compared to 415 nm is used to calculate the color change that occurs following the increase in the pH of the media, which is due to the urea hydrolysis by urease. Furthermore, the use of urease broth without urea takes into account any color change that is not due strictly to pH changes following urea hydrolysis, such as the release of other byproducts during growth, that occurs during the course of the experiment. Thus, the urease activity was calculated by dividing the OD values at 560 nm by the OD values at 415 nm and the urease activity was then normalized by dividing the OD of each strain grown in urease broth containing urea by the same strain grown in urease broth without urea.


$$Urease\;Activity=\frac{OD_{560}}{OD_{415}}$$

$$Normalized\;Urease\;Activity=\frac{Average\;Urease\;Activity(wells\;with\;urea)}{Average\;Urease\;Activity(wells\;without\;urea)}$$

### Statistical Analyses

Graphpad Prism 8.4.3 software was used to create all the normalized urease activity graphs. All experiments were performed with at least three replicates and repeated at least twice. Graphs display the mean of the combined replicates and error bars represent the standard deviation from the mean. An additional statistical analysis, using the Mann Whitney U Test, was performed on low urease producers to confirm urease activity detected among strains grown in the presence of urea was significantly different from that strain grown in the absence of urea.

## Results

### Urease Carriage

**Figure 2:**
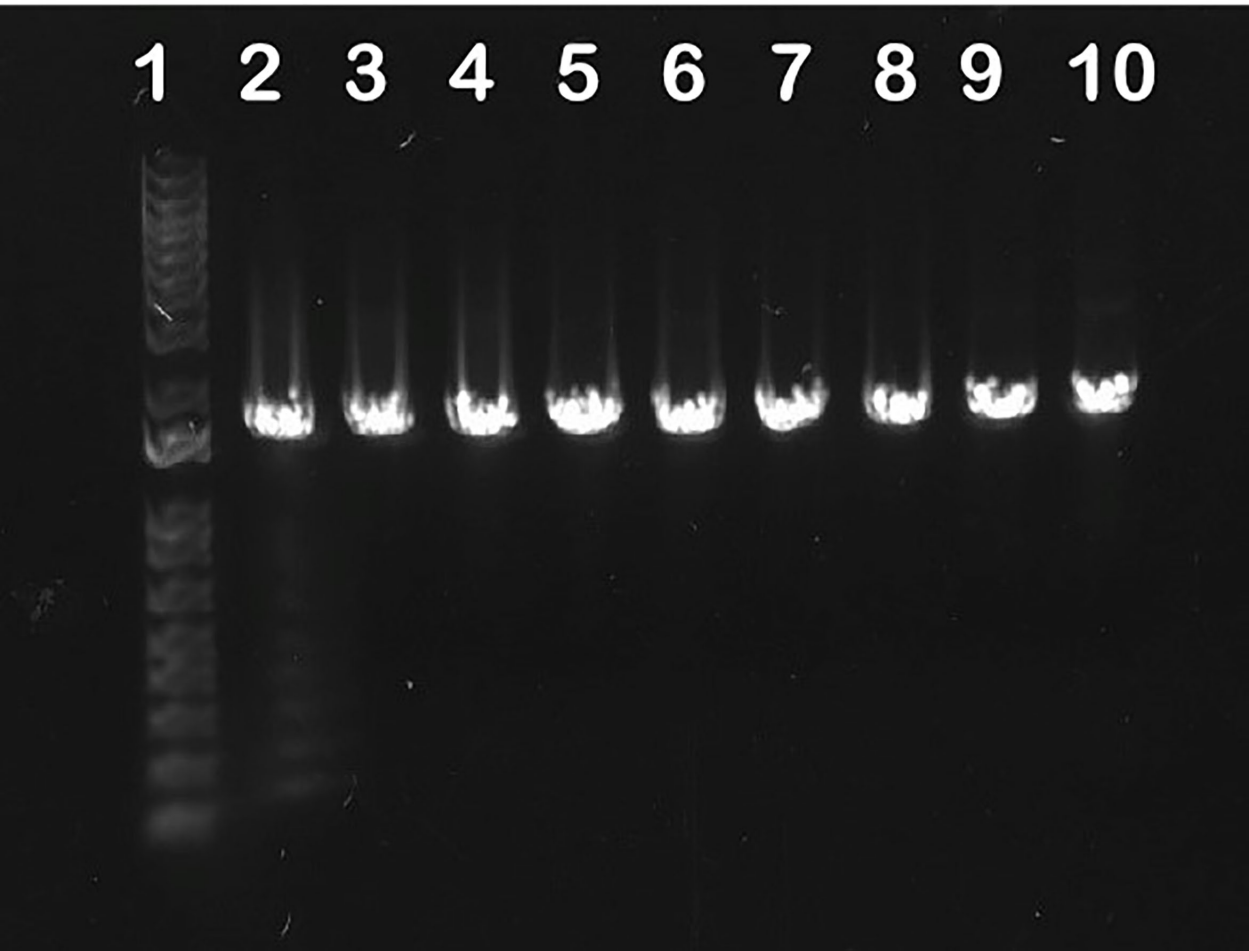
*S. aureus ureC* carriage. PCR using the ureC-Forward and ureC-Reverse primers (**Table 2**) was performed to confirm the presence of the *ureC* gene within clinical *S. aureus* isolates. The presence of an amplicon around 1.6 Kb indicates all the strains tested encoded *ureC*. Numbers denote lanes: (1) Ladder, (2) JE2, (3) UTI MRSA, (4) HUC 86, (5) HUC 86-02, (6) HUC 95, (7) HUC 97-02, (8) HUC 100, (9) HUC 100-01, (10) HUC 111-01C.

Previous reports indicate that urease detection among *S. aureus* clinical isolates is low (25%-50%) (Bichler et al., 2002; Gad et al., 2009; Flannigan et al., 2014). Yet a previous report suggested the urease operon was present in a majority (>90%) of sequenced isolates (Konieczna et al., 2012). Our analysis of 10,290 sequenced *S. aureus* isolates in NCBI found that *ureC* was present in 93.1% of these strains, supporting the previous report (Konieczna et al., 2012). Furthermore, to determine whether a similar proportion of our *S. aureus* human urinary catheter (HUC) isolates encoded urease, we used PCR to confirm the presence of the *ureC* gene, which is a marker for the operon. Amplicons corresponding to an ~1.6 Kb band indicate that the *ureC* gene was encoded by all HUC isolates (7/7), as well as the urine-associated strain UTI MRSA, and the skin infection isolate JE2 (**Figure 2**). Thus, all (9/9) *S. aureus* strains tested encoded *ureC*.

### Christensen Urea Agar Assay

**Figure 3:**
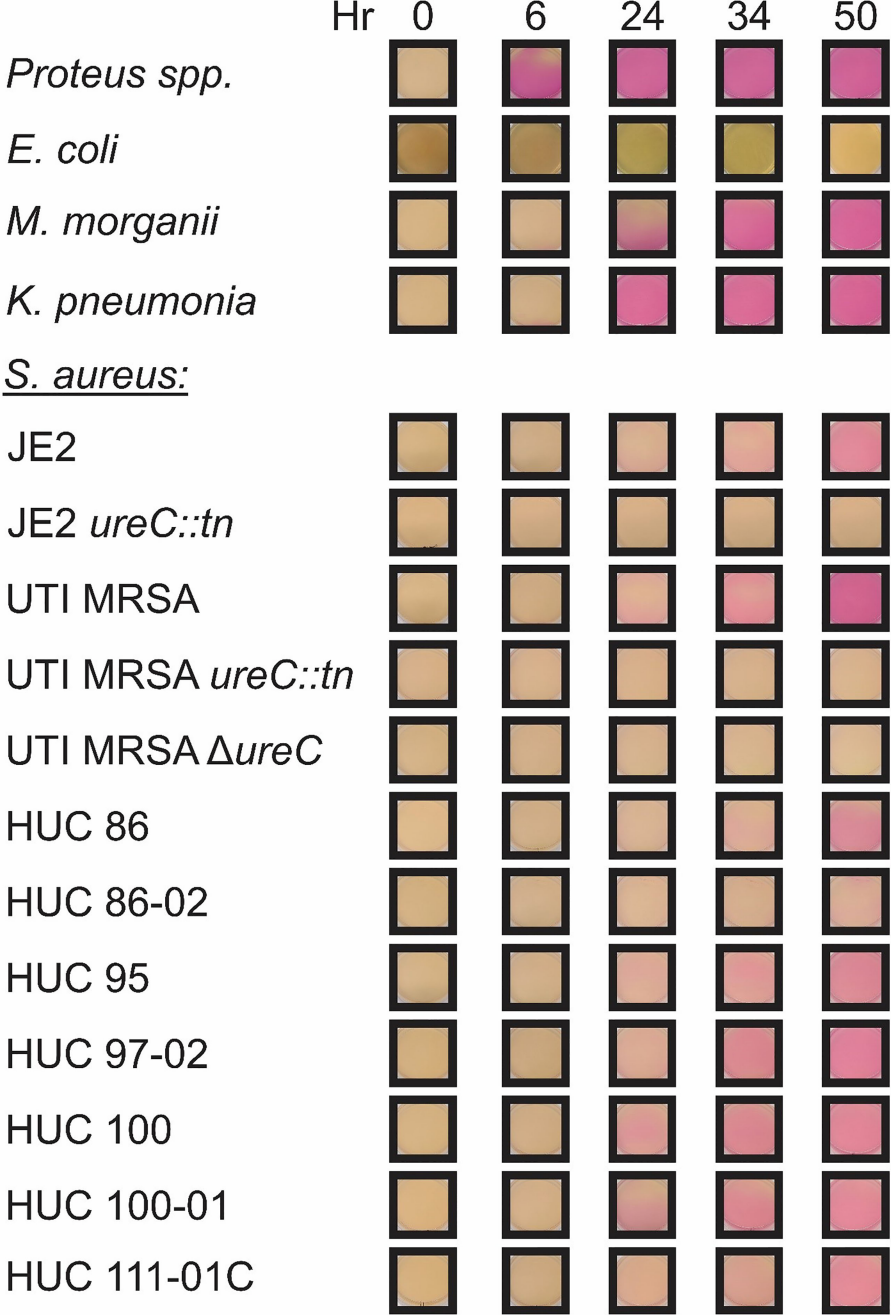
Representative assay of the Christensen Urea Agar urease activity test. Christensen Urea Agar plates were streaked with a single colony of each bacterial strain: *Proteus* spp.*, Escherichia coli, Klebsiella pneumonia, Morganella morganii*, and *Staphylococcus aureus* strains including JE2, JE2 *ureC::tn*, UTI MRSA, UTI MRSA Δ*ureC*, UTI MRSA *ureC::tn*, HUC 86, HUC 86-02, HUC 95, HUC 97-02, HUC 100, HUC 100-01, and HUC 111-01C. Urease activity was detected by 6 hours in *Proteus* spp., while no urease activity was detected in *Escherichia coli*. Urease activity was detected by 6 hours for both *Klebsiella pneumonia* and *Morganella morganii*. For *Staphylococcus aureus* strains, urease activity in was detected by 24 hours in UTI MRSA, HUC 95, HUC 97-02, HUC 100, and HUC 100-01, 34 hours in JE2, HUC 86, and HUC 111-01C, 50 hours in HUC 86-02. No urease activity was detected in JE2 *ureC::tn*, UTI MRSA Δ*ureC*, or UTI MRSA *ureC::tn*.

To determine whether urease could be detected in a diverse set of strains the current clinical test, the Christensen Urea Agar assay, was used (**Figure 3**). A *Proteus* spp. strain was used as a positive control and urease activity was rapidly detected, with the entire plate turning pink by 6 hours. *E. coli* was used as a negative, non-urease producing control and displayed no color change during the course of the experiment. *K. pneumonia* urease could be detected as early as 6 hours, with complete color change of the agar plate around 24 hours. *M. morganii* also displayed signs of urease activity as early as 6 hours, but complete color change of the agar plate was delayed compared to *K. pneumonia*, occurring around 34 hours. For the *S. aureus* strains, urease production could be detected as early as 24 hours for UTI MRSA, HUC 95, HUC 97-02, HUC 100, and HUC 100-01, 34 hours for JE2 and HUC 86, and 50 hrs for HUC 86-02. Thus, all (9/9) *S. aureus* strains tested were confirmed to be urease-producers by the Christensen Urea Agar method. All *S. aureus ureC* mutants remained negative, as they did not display any color change during the course of the experiment.

### Urease Broth Assay Validation

**Figure 4:**
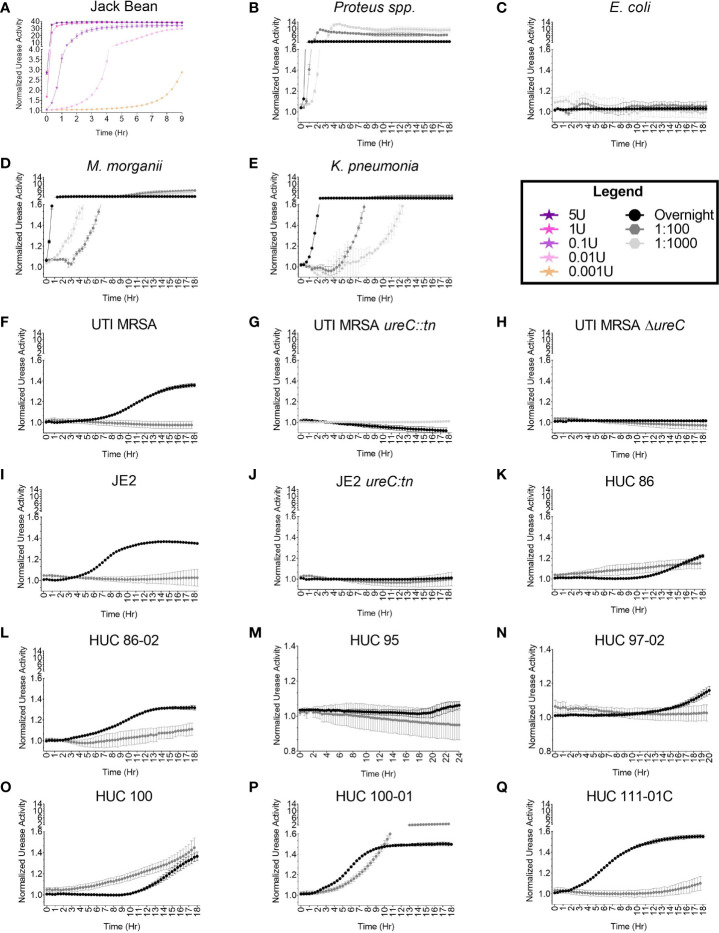
High throughput, semi-quantitative urease activity detection assay. The urease activity detection assay was validated with **(A)** Jack Bean enzyme, **(B)** *Proteus* spp., and **(C)** *Escherichia coli.* A dose response curve (5U, 1U, 0.1U, and 0.001U) of Jack Bean urease was assessed. Enzymatic activity was detected immediately for 5U and 1U, at 20 minutes for 0.1U, at 40 minutes for 0.01U, and at 4 hours for 0.001U. For the bacterial strains, increasing concentrations of bacteria were assessed including 1:1000 and 1:100 dilutions of overnight cultures, as well as overnight cultures resuspended in urease broth, with urease activity detected at 1.5 hours, 40 minutes, and 20 minutes for the *Proteus* spp. strain, respectively. The *E coli* strain did not display urease activity. Additional, diverse urease-producing uropathogens at 1:1000 and 1:100 dilutions of overnight cultures, as well as overnight cultures resuspended in urease broth were assessed for urease activity, including **(D)** *Morganella morganii* detected at 3 hours, 40 minutes, and 20 minutes, respectively; and **(E)** *Klebsiella pneumonia* detected at 4, 4, and 0.5 hours, respectively. For *S. aureus* strains urease activity was assessed at 1:100 dilutions of overnight cultures, as well as overnight cultures resuspended in urease broth, with **(F)** UTI MRSA detected at 4 hours only at the highest bacterial concentration (overnight suspension). The **(G)** UTI MRSA *ureC::tn* and **(H)** UTI MRSA Δ*ureC*, were used as negative controls. No urease was detected over the course of the 18-hour experiment. Furthermore, **(I)** JE2 urease was detected at 4 hours only in the highest bacterial concentration tested (overnight suspension), while no urease was detected for **(J)** JE2 *ureC::tn* across the course of the 18-hour experiment. Lastly, additional *S. aureus* strains were assessed for urease activity to determine the assay’s accuracy in detecting variable urease producers. Urease activity was detected for **(K)** HUC 86 at 10 hours and 3 hours for the 1:100 dilution and overnight culture suspension, respectively; **(L)** HUC 86-02 at 6 hours and 2 hours for the 1:100 and overnight suspension, respectively; **(M)** HUC 95 at 20 hours only in overnight suspensions; **(N)** HUC 97-02 at 12 hours only in overnight suspensions; **(O)** HUC 100 at 10 hours and 3 hours for the 1:100 dilution and overnight suspension, respectively; **(P)** HUC 100-01 at 4 hours and 1.5 hours for the 1:100 dilution and overnight suspension, respectively; and **(Q)** HUC 111-01C at 12 hours and 0.5 hour for the 1:100 dilution and overnight suspension, respectively. Points represent the mean of three technical replicates and two biological replicates and error bars represent standard deviation from the mean.

The adapted urease activity assay was evaluated using the positive controls: Jack Bean urease enzyme (**Figure 4A**) and a *Proteus* spp. strain (**Figure 4B**). The Jack Bean urease was used to create standard curves to assess activity with decreasing concentrations of the enzyme. The concentrations of pure Jack Bean included 5U, 1U, 0.1U, 0.01U, and 0.001U. Urease activity was detected immediately (within seconds) for the highest concentrations of Jack Bean (5U and 1U) in urea containing media. For the lower concentrations, a dose response was observed, with activity detected around 20 minutes for 0.1U, 40 minutes for 0.01U, and 4 hours for 0.001U (**Figure 4A**). Next, the urease activity of the *Proteus* spp. strain was assessed at various bacterial concentrations. For the highest concentrations of bacteria – overnight cultures resuspended in urease broth and cultures diluted 1:100 in urease broth – activity was detected around 20 and 40 minutes after the start of the assay, respectively (**Figure 4B**). For the lowest concentrations of bacteria – cultures diluted 1:1000 in urease broth – activity was detected around 1.5 hours after the start of the assay (**Figure 4B**). *E. coli* was used as a negative control. No urease activity was detected at any concentration over the course of the 18-hour assay (**Figure 4C**).

### Optimized Detection for Urease Producing Uropathogens

*M. morganii*, *K. pneumonia*, and *S. aureus* (UTI MRSA and JE2) strains, which are known urease producers, were selected to optimize the urease detection assay for diverse species. Urease activity was detected among various bacterial concentrations for each species and urease activity correlated with bacterial concentration (**Figure 4**). For *M. morganii*, urease activity was rapidly detected for the highest bacterial concentrations (overnight resuspensions), ~20 minutes after the start of the assay (**Figure 4D**). For lower bacterial concentrations of *M. morganii*, diluted 1:100 and 1:1000 in urease broth, enzymatic activity was detected about 40 minutes and 3 hours, respectively, after the start of the assay (**Figure 4D**). For *K. pneumonia*, urease activity could be detected as early as 40 minutes for the highest bacterial concentration (overnight resuspension) and at 4 hours for both the 1:100 and the 1:1000 dilutions (**Figure 4E**). For UTI MRSA, urease activity could be detected as early as 4 hours for the overnight concentration, while no color change was detected for the lower bacterial concentrations diluted 1:100 and 1:1000 (**Figures 4F**, **S1**). Furthermore, the UTI MRSA *ureC::tn* and UTI MRSA Δ*ureC* strains were used as non-urease producing negative controls and no urease was detected over the 18-hour time course (**Figures 4G, H**, **S1**). The well-characterized *S. aureus* skin infection isolate JE2 was also assessed in the adapted urease assay. Similarly to UTI MRSA, urease activity in JE2 was only detected at the highest bacterial concentration, with detection starting at 3 hours (**Figures 4I**, **S1**). JE2 *ureC::tn* was used as a non-urease producing negative control, and no urease was detected over the course of the 18-hour experiment (**Figures 4J**, **S1**).

### Assessing Urease-Producing *S. aureus* Clinical Isolates With Adapted Assay

A panel of clinical catheter-associated *S. aureus* isolates were selected to further assess the accuracy of the assay on so-called difficult to detect urease producers. For HUC 86, the adapted urease broth assay detected enzymatic activity around 10 hours at the highest bacterial concentration (overnight suspension) (**Figure 4K**). This contrasted with the Christensen Urea Agar plate assay, which detected urease activity around 34 hours. Furthermore, at the 1:100 bacterial concentration, the adapted urease assay detected some enzymatic activity with HUC 86 as early as 3 hours. No urease activity was detected with HUC 86 at the lowest dilution (1:1000) (**Figure S1**). Additionally, the adapted assay detected urease activity for the serial isolate from the same patient, HUC 86-02, more rapidly, around 2 hours at the highest bacterial concentration (overnight suspension) and at 6 hours for the 1:100 dilution (**Figure 4L**). No urease activity was detected at the lowest bacterial concentration (1:1000) (**Figure S1**). For the HUC 95 strain, urease activity was detected at 20 hours at the highest bacterial concentration (overnight suspension), and no urease activity was detected for the lower bacterial suspensions (1:100 or 1:1000) (**Figures 4M**, **S1**). For the HUC 97-02 strain, urease activity could begin to be detected at 12 hours at the highest bacterial concentration (overnight suspension), but no urease activity could be detected at the lower bacterial concentrations (1:100 or 1:1000) (**Figures 4N**, **S1**). For the HUC 100 strain, urease activity was first detected for the 1:100 dilution approximately 3 hours after the start of the assay (**Figure 4O**). Urease activity was next detected at 10 hours for the highest bacterial concentration (overnight suspension). No urease activity was detected at the lowest concentration (1:1000 suspension) (**Figure S1**). The HUC 100-01 strain, which is the serial isolate from the same patient as HUC 100, displayed the quickest urease activity at the highest bacterial concentration (overnight suspension) and was detected approximately 1 hour after the start of the assay. Notably, the lower bacterial concentration, 1:100, was also detected fairly rapidly, around 4 hours after the start of the assay (**Figure 4P**). No urease activity was detected at the lowest bacterial concentration, 1:1000 dilution (**Figure S1**). Furthermore, in contrast to the Christensen Urea Agar plate test, urease activity for HUC 111-01C was rapidly detected, as early as 40 minutes after the start of the assay, at the highest bacterial concentration (overnight suspension), with the lower bacterial concentration (1:100 dilution) exhibiting enzymatic activity around 12 hours (**Figure 4Q**). No urease activity was detected at the 1:1000 concentration (**Figure S1** **)**. Lastly, to provide additional analyses that further differentiate those isolates that display low activity from those that display no activity in the presence of urea, including *E. coli*, HUC 95, and HUC97-02, statistical analyses comparing the enzymatic activity in the presence and absence of urea were performed (**Figure S2**). The UTI MRSA strain was used as a positive control and displayed significantly more urease activity at the final time point assessed when grown in the presence of urea compared to the absence, while no difference was detected in the UTI MRSA *ΔureC* strain between the two conditions (**Figures S2A, B**). Additionally, significantly more urease activity was detected for both HUC 95 and HUC 97-02 when the strains were grown with urea compared to without (**Figures S2C, D**). Lastly, *E. coli* displayed no difference in detected urease activity when the bacteria were grown in the presence of urea compared to in its absence at the final time point assessed, while the *Proteus* spp. strain grown with urea had significantly more urease activity detected compared to the condition without urea (**Figures S2E, F**).

### Growth Curves of Urease-Producing HUC Isolates in the Adapted Assay

To assess whether urease-producing uropathogens could grow in the adapted assay media, bacterial growth was measured at an OD_600_ (**Figure S3**). *Proteus spp, E. coli, M. morganii, and K. pneumonia* all displayed similar growth phenotypes, with increasing growth observed within the first few hours of the assay for all bacterial concentrations, before plateauing **(** **Figures S3A–D**). The UTI MRSA and the isogenic *ureC::tn* and *ΔureC* strains displayed slight increased growth during the first few hours at the highest bacterial concentration (overnight suspensions); however, no growth was observed over the time course of the experiment for the 1:100 and 1:1000 bacterial concentrations (**Figures S3E–G**). For the JE2 and the isogenic *ureC::tn*, no growth was detected for any of the bacterial concentrations over the course of the experiment (**Figures S3H–K**). The HUC 86 and HUC 86-02 displayed slight increased growth when diluted 1:100; however, no growth was observed for the overnight or 1:1000 bacterial concentrations (**Figures S3J, K**). The HUC 95 strain displayed growth at the highest bacterial concentration in the last few hours of the experiment; however, no growth was observed for the 1:100 and 1:1000 bacterial concentrations (**Figures S3L**). Additionally, no growth was detected for HUC 97-02 at any bacterial concentration over the course of the experiment (**Figure S3M**). Steadily increasing growth was detected in the HUC 100 and HUC 100-01 over the course of the experiment for the 1:100 bacterial concentration; however, no growth was detected for overnight and 1:1000 bacterial concentrations (**Figure S3N, O**). Lastly, HUC 111-01C displayed increased growth within the first few hours of the assay at the 1:1000 bacterial concentration, but no growth was detected for overnight and 1:100 bacterial concentrations (**Figure S3P**).

## Discussion

Over 1 million cases of CAUTIs occur each year (Ronald, 2003; Trautner and Darouiche, 2004; Cope et al., 2009; Trautner, 2010; Nicolle, 2012; Flores-Mireles et al., 2015; Chenoweth and Saint, 2016). Unlike uncomplicated UTIs in healthy individuals, CAUTIs are caused by a broader range of pathogens that continue to be understudied (Konieczna et al., 2012; Wagenlehner et al., 2012; Weiner et al., 2016). In addition, chronically catheterized individuals are at a high risk for ASB, which when caused by urease-producing bacteria can result in catheter blockages, pyelonephritis, and lead to a decrease in quality of life (Warren et al., 1994; Hedelin, 2002; Broomfield et al., 2009; Flores-Mireles et al., 2015; Nicolle, 2019). With the projected increase in urinary catheterizations due to an aging population, the importance of understanding urease activity among agents other than *P. mirabilis* is apparent, as urease production is a key component causing catheter encrustations that greatly reduce the efficacy of catheters, decrease the quality of life of patients, and increase the risk of UTIs (Broomfield et al., 2009; Trautner, 2010). However, current assays to detect urease activity favors high urease producers like *P. mirabilis*. Specifically, our data from Christensen Urea Agar plates demonstrate that rapid detection only works well for *Proteus* spp. The diversity of urease-producing uropathogens highlights the importance of developing an assay that can detect urease activity for a broader range of microbes that may require different culture conditions. Furthermore, the current clinical assays are primarily designed for qualitative detection of urease activity, leaving little room for more complex studies focused on regulation, production, or activity.

We developed a versatile, high throughput assay to allow us to detect urease activity among diverse urease-producing CAUTI pathogens. We validated the assay utilizing Jack Bean urease enzyme and *Proteus* spp. as positive controls and *E. coli* as a negative control. Importantly, when compared to the Christensen Urea Agar plate method, the adapted assay more rapidly detected urease activity for a wide range of species, including *Proteus* spp., *K. pneumonia*, *M. morganii*, and *S. aureus*. Furthermore, while all clinical *S. aureus* strains tested were confirmed to encode *ureC*, urease activity in these isolates was highly variable. Specifically for the adapted urease assay, no urease activity was detected at the lowest concentration (1:1000 dilution), yet at the 1:100 dilution around 55% (5/9) of *S. aureus* strains were identified as urease producers. This percentage matches what is typically reported in the clinical setting, indicating a 1:100 dilution may mimic the results from the current Christensen Urea Agar method. However, our PCR results indicate all *S. aureus* strains encode urease and our adapted assay more closely replicated those results using the highest bacterial concentration. Furthermore, our adapted assay more quickly detected urease activity in UTI MRSA and JE2, at 4 hours and 3 hours, compared to the Christensen Urea Agar method, which detected activity at 24 hours and 34 hours, respectively. Additionally, urease activity for the HUC isolates, HUC 86, HUC 86-02, HUC 95, HUC 97-02, HUC 100, HUC 100-01, and HUC 111-01C, was detected at 10, 2, 20, 12, 3, 1.5, and 0.5 hours with the adapted assay compared to the Christensen Urea Agar method, which detected activity at 34, 50, 24, 24, 24, 24, and 34 hours, respectively.

While studies investigating urease regulation among “weak” urease producers remains limited, two recent studies have indicated *S. aureus* urease may be regulated *via* several pathways, including the carbon catabolite pathway, CcpA, the quorum sensing system, Agr, and a global regulator, CodY (Seidl et al., 2009; Zhou et al., 2019). Specifically, for the Agr system in *S. aureus*, activation is cell density dependent, with expression of the system occurring at high bacterial concentrations due increased quantities of the autoinducing peptide (AIP) (Zhou et al., 2019). Once a threshold of AIP is reached, Agr upregulates the expression of downstream genes, including urease. Notably, through this adapted urease assay, a density dependent relationship was detected with higher bacterial load correlating with higher urease activity and quicker detection among the *S. aureus* strains. This supports the previous study that indicated the agr quorum sensing system may be involved in urease regulation. Furthermore, it may explain why some variability was detected even among the highest bacterial concentration tested, specifically since urease was detected in HUC 95 only after growth occurred at the latest time points.

In addition to shortening the time of detection for urease activity, our adapted assay also detected noticeable differences in urease activity among the sequential HUC isolates. Specifically, the adapted assay detected urease activity for the each of the sequential isolates more quickly compared to the initial isolates (HUC 86-02 vs HUC 86 and HUC 100-01 vs HUC 100). This is in contrast to the Christensen Urea Agar method, which indicated the HUC 100 and HUC 100-01 isolates exhibited similar urease activity, while HUC 86 produced more urease activity compared to the sequential isolate HUC 86-02. Together, our data suggests that chronic urinary catheterization may select for isolates with increased urease activity. However, this is a small sample size and should be assessed in future studies in more detail.

Importantly, the adapted assay allows for the manipulation of various conditions to assess different aspects of urease production. Specifically, we were able to alter the concentration of bacteria to assess urease activity and production at different growth stages and among diverse species of bacteria. While there are limitations to this modified assay, they are similar to those for the other clinical assays, including the Christensen Urea Agar method and the RUT test. Specifically, this assay is also limited by the time the assay can accurately detect urease activity due to the risk of false positives caused by bacterial growth byproducts or other non-urease related pH changes in the media. However, the inclusion of non-bacteria containing controls, non-urea containing controls, and non-urease producing strains are essential for mitigating these limitations. Additionally, the current composition and conditions may need to be further adjusted to test for other uropathogens or to accommodate for other, as yet unknown environmental stimuli or factors that affect urease regulation among these diverse species. Since the assay is easily adaptable, these adjustments can be made using validated controls. Thus, this adaptable, high throughput, semi-quantitative assay can facilitate future studies that dissect differences in urease regulation or activity in a broad range of urease-producing pathogens by modifying the culture conditions. By studying the regulation of the urease enzyme, better treatments and/or potential therapies can be developed to potentially reduce CAUTIs, and improve the quality of life for catheterized individuals.

## Data Availability Statement

The raw data supporting the conclusions of this article will be made available by the authors, without undue reservation.

## Author Contributions

JD: project development, data collection and analysis, and manuscript writing and editing. JG: data collection and analysis, and manuscript writing. CO: mutant generation and data collection. NG: mutant generation and data collection. JW: data collection and analysis, project development, and manuscript writing and editing. All authors contributed to the article and approved the submitted version.

## Funding

Research reported in this publication was supported by the NIH NIDDK under Award Number 1K01DK128381 and by funds from the University of Texas Health Science Center at Houston, as well as the Rising Star Award from the University of Texas system.

## Conflict of Interest

The authors declare that the research was conducted in the absence of any commercial or financial relationships that could be construed as a potential conflict of interest.

## Publisher’s Note

All claims expressed in this article are solely those of the authors and do not necessarily represent those of their affiliated organizations, or those of the publisher, the editors and the reviewers. Any product that may be evaluated in this article, or claim that may be made by its manufacturer, is not guaranteed or endorsed by the publisher.
