# Supplementary material for: Semi-Quantitative Assay to Measure Urease Activity by Urinary Catheter-Associated Uropathogens
Source: Front Cell Infect Microbiol. 2022 Mar 22;12:859093. doi: 10.3389/fcimb.2022.859093 (PMC8980526; doi:10.3389/fcimb.2022.859093)
Supplement: Supplementary file 1 [file Table_1.docx]

Supplementary Material

**Supplementary Figures**


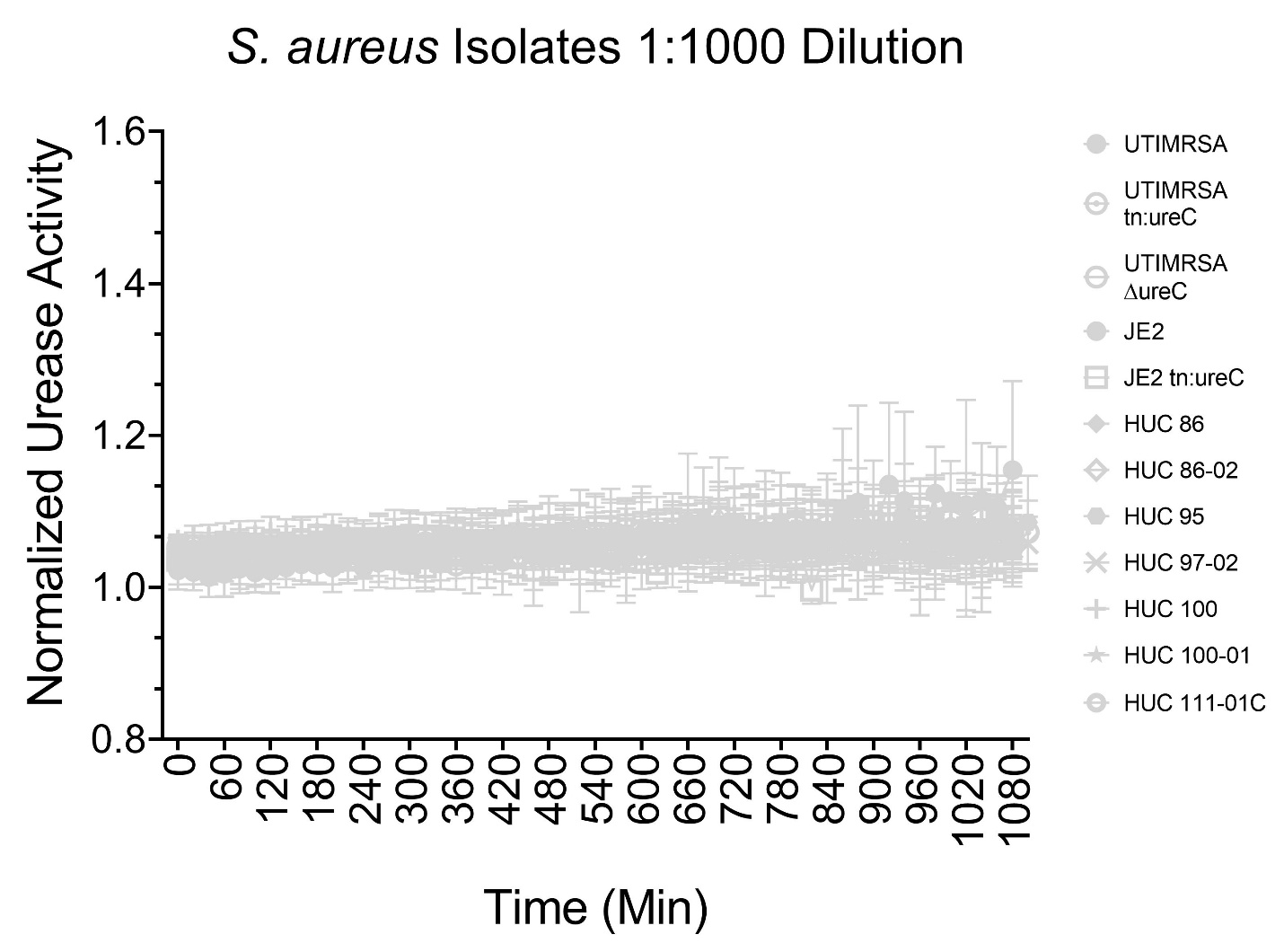
**Figure S1. Urease activity among *S. aureus* Isolates diluted 1:1000.** No urease activity was detected in UTI MRSA, UTI MRSA *ureC::tn*, UTI MRSA *ΔureC*, JE2, JE2 ureC::tn, HUC 86, HUC 86-02, HUC 95, HUC 97-02, HUC 100, HUC 100-01, and HUC 111-01C when the bacteria were tested at low bacterial concentrations.


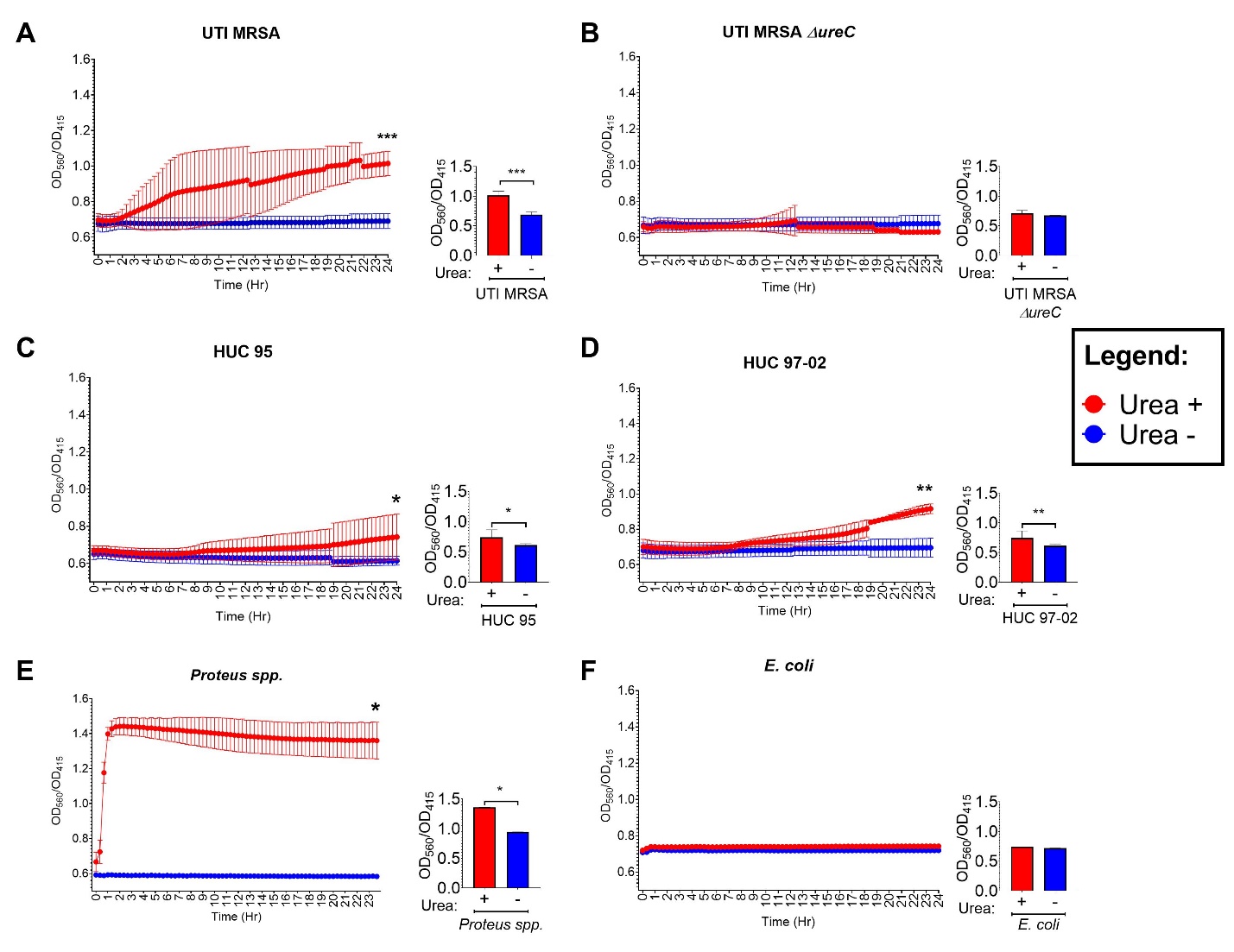


**Figure S2. Statistical analyses of urease activity among urease-producing *S. aureus* strains.** Analyses were performed for each end point of the urease activity assay to determine whether enzymatic activity detected among strains grown in the presence urea was significantly different from those grown in the absence of urea. The OD_560/415_ ratio was plotted over time for A) UTI MRSA B) UTI MRSA *ΔureC*, C) HUC 95, D) HUC 97-02, E) *Proteus spp.*, and F) *E. coli*. At the final time point assessed, UTI MRSA, HUC 95, HUC 97-02, and *Proteus spp.* grown in the presence of urea had significantly more urease activity detected compared to the same strain grown without urea. No difference was detected in urease activity among the UTI MRSA *ΔureC* or *E. coli* strains grown with or without urea. Mann Whitney U test was used to determine statistical significances, with *=p<0.05, **=p<0.005, and ***=p<0.0001.


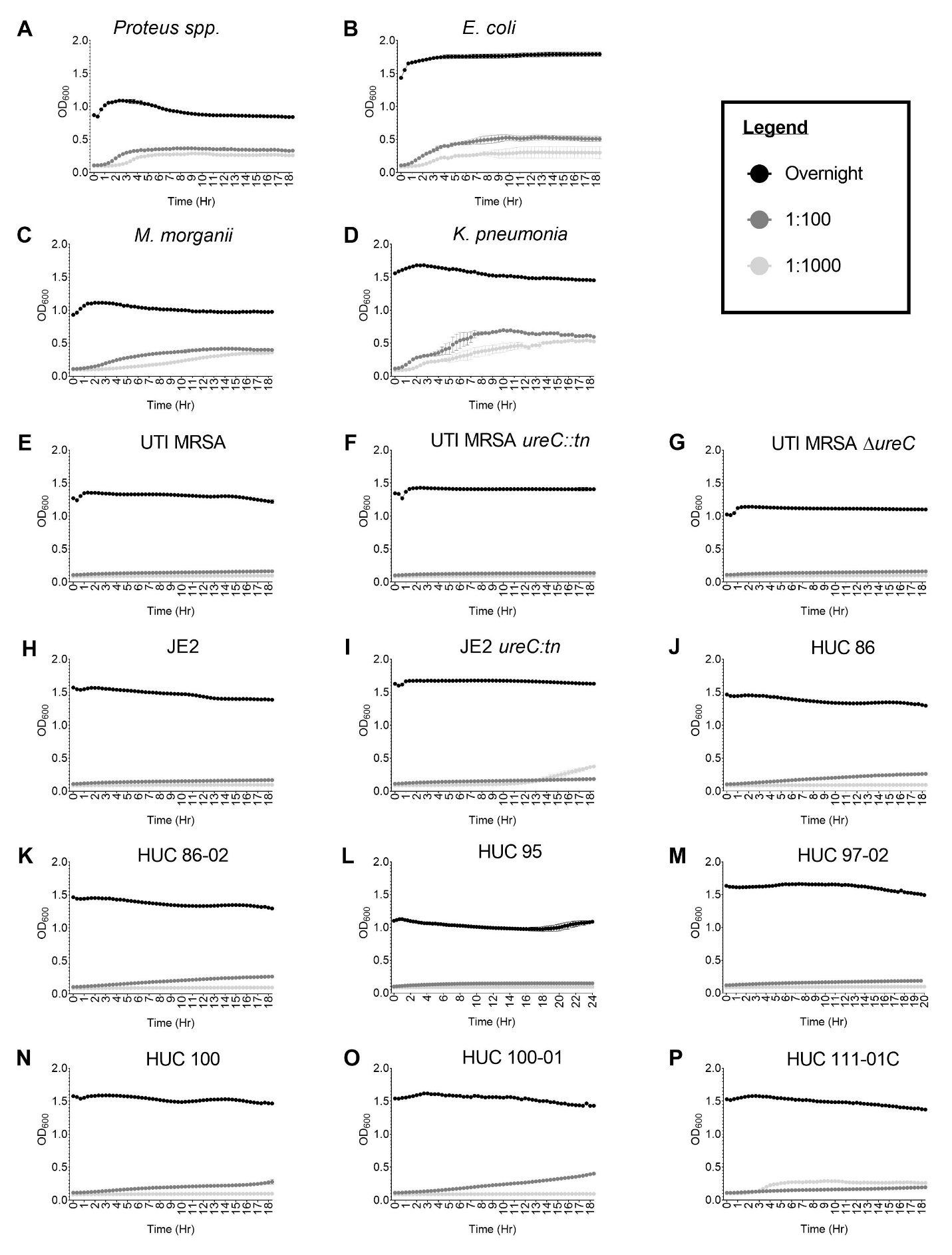


**Figure S3. Growth curves of all uropathogen isolates tested for urease activity.** Bacterial growth in the adapted urease activity assay was assessed at an OD_600_ for all bacterial concentrations tested. Growth was observed in the first few hours of overnight, 1:100, and 1:1000 bacterial concentrations for (A) *Proteus spp.*, (B) *Escherichia coli*, (C) *Morganella morganii,* and (D) *Klebsiella pneumonia* before plateauing for the rest of the experiment*.* For (E) UTI MRSA, (F) UTI MRSA *ureC::tn*, and (G) UTI MRSA *ΔureC*, initial growth was observed during the first few hours in the overnight bacterial concentration, while no growth was observed over the time course of the experiment for the 1:100 and 1:1000 bacterial concentrations. For (H) JE2 and (I) JE2 *ureC::tn* no growth was detected for the overnight, 1:100, or 1:1000 bacterial concentrations over the course of the experiment. For (J) HUC 86 and (K) HUC 86-02 no growth was detected for the overnight or 1:1000 concentrations; however, slight increasing in growth were observed for the 1:100 dilutions. For (L) HUC 95 while growth was detected for the overnight bacterial concentrations near the end of the experiment, no growth was observed for the 1:100 and 1:1000 bacterial concentrations. Additionally, no growth was detected for the (M) HUC 97-02 overnight, 1:100, or 1:1000 bacterial concentrations. For (N) HUC 100 and (O) HUC 100-01 steady growth was observed over the course of the experiment for the 1:100 bacterial concentration, while no growth was detected for overnight and 1:1000 bacterial concentrations. For (P) HUC 111-01C the 1:1000 bacterial concentration displayed increased growth within the first few hours of the assay, but no growth was detected for overnight and 1:100 bacterial concentrations.
